# Endothelium Trans Differentiated from Wharton's Jelly Mesenchymal Cells Promote Tissue Regeneration: Potential Role of Soluble Pro-Angiogenic Factors

**DOI:** 10.1371/journal.pone.0111025

**Published:** 2014-11-20

**Authors:** Valeria Aguilera, Luis Briceño, Hector Contreras, Liliana Lamperti, Esperanza Sepúlveda, Francisca Díaz-Perez, Marcelo León, Carlos Veas, Rafael Maura, Jorge Roberto Toledo, Paulina Fernández, Ambart Covarrubias, Felipe Andrés Zuñiga, Claudia Radojkovic, Carlos Escudero, Claudio Aguayo

**Affiliations:** 1 Department of Clinical Biochemistry and Immunology, Faculty of Pharmacy, University of Concepción, Concepción, Chile; 2 Faculty of Medicina, Universidad Católica de la Santísima Concepción, Concepción, Chile; 3 Department of Physiopathology, Faculty of Biological Sciences, University of Concepción, Concepción, Chile; 4 Facultad Ciencias de la Salud, Escuela de Tecnología Médica, Universidad San Sebastián, Concepción, Chile; 5 Vascular Physiology Laboratory, Group of Investigation in Tumor Angiogenesis (GIANT), Department of Basic Sciences, University of Bío-Bío, Chillán, Chile; 6 Group of Research and Innovation in Vascular Health (GRIVAS Health), Department of Basic Sciences, University of Bío-Bío, Chillán, Chile; University of Udine, Italy

## Abstract

**Background:**

Mesenchymal stem cells have a high capacity for trans-differentiation toward many adult cell types, including endothelial cells. Feto-placental tissue, such as Wharton's jelly is a potential source of mesenchymal stem cells with low immunogenic capacity; make them an excellent source of progenitor cells with a potential use for tissue repair. We evaluated whether administration of endothelial cells derived from mesenchymal stem cells isolated from Wharton's jelly (hWMSCs) can accelerate tissue repair *in vivo*.

**Methods:**

Mesenchymal stem cells were isolated from human Wharton's jelly by digestion with collagenase type I. Endothelial trans-differentiation was induced for 14 (hWMSC-End14d) and 30 (hWMSC-End30d) days. Cell phenotyping was performed using mesenchymal (CD90, CD73, CD105) and endothelial (Tie-2, KDR, eNOS, ICAM-1) markers. Endothelial trans-differentiation was demonstrated by the expression of endothelial markers and their ability to synthesize nitric oxide (NO).

**Results:**

hWMSCs can be differentiated into adipocytes, osteocytes, chondrocytes and endothelial cells. Moreover, these cells show high expression of CD73, CD90 and CD105 but low expression of endothelial markers prior to differentiation. hWMSCs-End express high levels of endothelial markers at 14 and 30 days of culture, and also they can synthesize NO. Injection of hWMSC-End30d in a mouse model of skin injury significantly accelerated wound healing compared with animals injected with undifferentiated hWMSC or injected with vehicle alone. These effects were also observed in animals that received conditioned media from hWMSC-End30d cultures.

**Conclusion:**

These results demonstrate that mesenchymal stem cells isolated from Wharton's jelly can be cultured in vitro and trans-differentiated into endothelial cells. Differentiated hWMSC-End may promote neovascularization and tissue repair *in vivo* through the secretion of soluble pro-angiogenic factors.

## Introduction

Mesenchymal cells (MSCs) are a heterogeneous group of adult stem cells able to proliferate and differentiate into osteocytes, chondrocytes or adipocytes [1], [2], as well as into non mesodermal precursors, from which they can be trans-differentiated towards hepatocytes [3], neurons [4] or endothelial cells [5], [6]. In addition, MSCs are multipotent cells with a potential use in human regenerative medicine due to their ability to migrate into sites of injury and modulate immune response. MSCs have been proposed for therapeutic use in degenerative diseases of bone, muscle, nervous system and specially for cardiovascular and heart diseases [7]–[9] but one of the main limitations is the quantity of cells that can be isolated from sources such as bone marrow [5]. However MSCs can also be obtained from other tissues, such as gingival tissue [10], skin [11], placenta [12], [13], amniotic fluid [14], whole blood or Wharton's jelly from umbilical cords [15], [16].

Mesenchymal stem cells isolated from Wharton's jelly (hWMSCs) have high telomerase activity [17], high proliferative capacity [17] and long- term culturing of expanded [18]–[20]. These cells cannot undergo tumor transformation [20]–[23] and have a low expression of histocompatibility complex class I molecules [18], [19], [22], while they do not express major histocompatibility complex class II [18], [22], [24]. Therefore, these cells exhibit low immunogenicity and high immunosuppressive capacity that make them useful for therapeutic approaches [25], [26].

*In vivo* experiments showed that hWMSCs can repair ischemic tissue by promoting neovascularization [27] and re-endothelialization [27]. The underlying mechanisms associated with pro-angiogenic effect of both MSCs and hWMSCs are under investigation. Indeed, MSCs can secrete angiogenic factors [27], and therefore, increase neovascularization in a mouse model of ischemia [28]. Indeed, MSCs can be differentiated into endothelial cells and form capillary-like structures, an effect associated with the production of vascular endothelial growth factor (VEGF) by those cells [29]–[31]. Moreover, MSCs increased neovascularization and tissue perfusion through the secretion of VEGF and stromal cell-derived factor 1 (SDF-1) [32], [33]. Despite these evidences there is limited information regarding potential pro-angiogenic activity of hWMSCs. Therefore we aimed to evaluate whether administration of endothelium derived from mesenchymal stem cells isolated from Wharton's jelly (hWMSCs) can accelerate tissue repair *in vivo*.

## Materials and Methods

### Ethics statement

This investigation conforms to the principles outlined in the Declaration of Helsinki, and has received approval from the Ethics Committee of the Faculty of Pharmacy of Universidad de Concepción de Chile and Guillermo Grant Benavente Hospital (Concepción, Chile). Informed written consent was obtained from all patients.

#### Isolation and culture of hWMSC

Umbilical cords were aseptically collected from healthy full-term pregnancies, at Guillermo Grant Benavente Hospital (Concepción, Chile). hWMSCs were isolated as described previously [27] with some modifications. Umbilical cords were cut into 2 to 3 mm^3^ pieces and vessels were stripped manually from those cord segments. Wharton's jelly was digested with collagenase 10 mg/mL (37°C for 4 hours). Mesenchymal cells were recovered, centrifuged (1000 g for 30 minutes) and suspended in fresh M-199 medium including 10% fetal bovine serum (FBS) and 5 ng/mL basic fibroblast growth factor, bFGF (Sigma, Aldrich). Cultures were maintained in a humidified atmosphere with 5% CO_2_ at 37°C for 10 days, until hWMSCs colonies were observed. Adherent cells were detached using a trypsin-EDTA solution [27].

#### hWMSCs differentiation

To confirm hWMSCs functionality, they were differentiated into osteocytes, adipocytes or chondrocytes. Cells were cultured in a 24-well plate at a density of 10×10^3^ cell/cm^2^ and when they reached about 70% confluence, culture medium was replaced with M-199 medium containing 10% FBS and 2 mM glutamine, and supplemented as follows to induce either osteogenic differentiation (0,1 µM dexamethasone, 50 µg/ml ascorbic acid, and 10 mMβ-glycerophosphate), adipogenic differentiation (0,1 µM dexamethasone, 60 µM Indomethacin, 0,5 mM 3-isobutil-1- metilxantine and 10 µg/mL insulin) or chondrogenic differentiation (0,1 µM dexamethasone, 10 ng/mL transforming growth factor β1). Cells were incubated for 15 days and media was changed every 3 days. Osteogenic differentiation was analyzed by von Kossa staining, adipogenic differentiation was confirmed by neutral lipid vacuoles detected with Oil Red O staining [27] and chondrogenic differentiation was verified by extracellular matrix staining with Alcian blue [24], [27], [34].

#### Flow Cytometry

Cell characterization by flow cytometry was carried out as previously described [19], [20], [27]. In brief, hWMSCs in culture were trypsinized (0.25% trypsin/1 mM EDTA) washed and resuspended in phosphate-buffered saline at a density of 3×10^5^ cells/100 µL. Cells were stained with an anti-human CD90-FITC monoclonal antibody (1∶100, Becton Dickinson, San Diego, CA, USA). Fifty thousand events were analyzed per sample in an argon laser FACS Calibur (BD Biosciences, Pharmingen, San Diego, CA, USA), at the Laboratory of Molecular Biology, Faculty of Medicine, Catholic University of Concepción.

#### Quantitative PCR

Total RNA was isolated using the Trizol Reagent (Invitrogen, Carlsbad, CA, USA) according to the manufacturers instructions. RNA quality and integrity and quality were insured by gel visualization and spectrophotometric analysis (OD_260/280_). 1 µg of total RNA was reversed transcribed into cDNA for 1 hour at 37°C as described elsewhere. [35]–[37].

mRNA for CD90, CD73, CD105 and Cyclophilin were assessed by quantitative real time PCR (qPCR) in a Rotorgene 2000 thermal cycler (Corbett Research). Thus, reactions in 25 µL were carried out using Brilliant II SYBR Green QPCR Master Mix (Agilent Technologies, USA) with 0.3 µM primers according to the manufacturer's instruction. Primers used are described in Table 1. Samples were incubated for 4 minutes at 95°C, followed by 25 cycles of 30 seconds at 95°C, 30 seconds at 54°C (for eNOS and Cyclofilin), 58°C (CD90, CD105) or 60°C (CD73), 30 seconds at 72°C, and finally 7 minutes at 72°C. A single melting point was observed for all samples. The gene expression was quantified using the 2−ΔΔCt (threshold cycle) method. Thus, a delta C(T) (ΔCt) value was obtained by subtracting the cyclophilin-CT value from the CT value of the studied gene. The ΔCt mean value obtained from the control group of each gene was used to calculate the 2(-Delta Delta C(t) (ΔΔCt) of the respective gene (2−ΔΔCt). Expected size products were separated by electrophoresis on 1.5% agarose gels and visualized with ethidium bromide under UV light.

**Table 1:** Oligonucleotide Primers for Reverse Transcriptase–Polymerase Chain Reaction.

**Cyclophilin**	**Sense**	**F-5′- CTCTTCGCCGATACCACTCC -3′**
	Antisense	R-5′- TCACACGGTGGAAGGTTGAG-3′
CD90	Sense	F- 5′-TTTGGCCCAAGTTTCTAAGG-3′
	Antisense	R- 5′-AGATGCCATAAGCTGTGGTG-3′
CD105	Sense	F- 5′-TCCAGCACTGGTGAACTGAG-3′
	Antisense	R- 5′-TGTCTCCCCTGCCAGTTAGT-3′
CD73	Sense	F-5′- CCTGCTCAGCTCTGCATAAGTA -3′
	Antisense	R-5′- CCCTATTTTACTGGCCAAGTGT -3′
CD31	Sense	F-5′-CAACAGACATGGCAACAAGG-3′
	Antisense	R-5′-TTCTGGATGGTGAAGTTGGC-3′
CD34	Sense	F-5′-AATGAGGCCACAACAAACATCACA-3′
	Antisense	R-5′-CTGTCCTTCTTAAACTCCGCACAGC-3′
eNOS	Sense	F-5′-CCAGCTAGCCAAAGTCACCAT-3′
	Antisense	R-5′-GTCTCGGAGCCATACAGGATT-3′
KDR	Sense	F- 5′-AGACCAAAGGGGCACGATTC-3′
	Antisense	R- 5′-GTCTGGTCTTTTGGTGTTTTGCTG-3′

### Immunocytochemistry

Staining was performed on fixed cell monolayers (4% paraphormaldehyde, 10 min, room temperature), which were grown previously on coverslips. hWMSCs were permeabilized with PBS 0.25% Triton X-100 for 10 min and antibodies were added overnight at 4°C (anti-vimentin (1∶500, Abcam, Cambridge, UK), CD31-FITC (1∶200, BD Pharmingen, San Diego, CA, USA), and anti-KDR-FITC (1∶300, BD Pharmingen, San Diego, CA, USA). For vimentin labeling, a secondary Texas-Red conjugated antibody was used. Samples were placed on slides using mounting medium and DAPI was added for nuclei identification. Microscopic analysis of samples was performed by using an Olympus IX81 inverted microscope in conjunction with a DSU spinning disk confocal system.

#### Endothelial differentiation

Endothelial differentiation was performed as described previously with some modification [5], [37]. hWMSCs were incubated in 100 mm^2^ plates up to 14 (hWMSC-End14d) or 30 days (hWMSC-End30d) in primary culture medium (PCM) composed of endothelial growth medium (GIBCO BRL Life Technologies, Bethesda, MD, USA) containing 5 mM D-glucose, 15% fetal calf serum, 10 mg/mL of human VEGF (GIBCO BRL Life Technologies, Bethesda, MD, USA) [35]–[37]. Endothelial phenotype was confirmed through presence of endothelial markers and nitric oxide (NO) production as described below.

#### Endothelial nitric oxide synthase (eNOS) activity

L-[^3^H]-arginine conversion into L-[^3^H]-citruline was used to assess the endothelial nitric oxide synthase (eNOS) activity in hWMSCs trans-differentiated into endothelial cells. hWMSC, hWMSC-End14d or hWMSC-End30d were incubated with Na^+^-Krebs solution containing 100 µM L-arginine and L-[^3^H]arginine (4 µCi/mL) for 30min at 37°C. Cells were lysed and treated in a cation ion-exchange resin Dowex-50W (50X8-200, Na^+^ form), to separate L-[^3^H]-citrulline present in H_2_O eluates from L-[^3^H]-arginine retained in the column. When required, N^G^-nitro-L-arginine methyl ester (L-NAME, 100 µM), a NOS inhibitor, was added to cell cultures 30 min before experiments [36].

#### Intracellular NO formation

Intracellular NO production was determined using the fluorescent probe 4-amino-5-methylamino-2′, 7′-difluorofluorescein diacetate (DAF-FM-DA, Calbiochem) as described previously [33], [37]. hWMSCs, hWMSC-End14d or hWMSC-End30d were seeded in 96-well plates, incubated for 24 h under standard conditions. Before experiments, cells were incubated in serum-depleted medium for 4 h. DAF-FM-DA probe (5 µM) and L-arginine (100 µM) were added to cultures for 30 minutes and then fluorescence intensity was measured in a fluorimeter (Sinergy 2, Biotec). As a positive control, 1 mM histamine was added for the last 15 min. To assess the involvement of NOS activity in NO production, cells were pre-incubated with L-NAME (100 µM, 30 min).

#### eNOS and vimentin protein expression

Total eNOS and vimentin protein expression was evaluated by western blot in hWMSCs, hWMSC-End14d or hWMSC-End30d. Total proteins (50 µg) were separated by a polyacrylamide gel (10%) electrophoresis and transferred to Immobilon-P polyvinylidenedifluoride membranes (BioRad Laboratories, Hertfordshire, UK). Membranes were probed with rabbit anti-eNOS (1∶1000), anti-vimentin (1∶500) or anti-β actin (1∶2000) (Santa Cruz Biotechnology, CA, USA). Membranes were incubated (1 h) with a horseradish peroxidase-conjugated goat anti-rabbit antibody. Proteins were detected by the enhanced chemiluminescence method, analyzed by densitometry and compared to β-actin expression (control).

#### Wound healing model and hWMSC transplantation

Seventy- C57BL-6 mice (6 weeks old; female; body weight 20–23 g) were randomly divided into four groups, (n = 5 per group), and the excisional wound-splinting model was generated as described previously [38]–[40]. The experimental protocols were approved by Ethics Committee of the Universidad del Concepción and conform to the guidelines for the Care and Use of Laboratory Animals published by the US National Institute of Health. In brief, after anesthesia and hair removal from the dorsal surface, two 6-mm full thickness excisional skin wounds were created on each side of the midline. Each wound received 5×10^5^cells (either, hWMSCs, hWMSC-End140d or hWMSC-End30d, in duplicate, each set derived from the same hWMSC line, with 5 hWMSC lines in total) in 60 µl of PBS injected intradermally around the wound at four injection sites; or 60 µl of conditioned media (see below), or PBS (control group). 20 µl of growth factor-reduced Matrigel (BD Biosciences) were applied onto the wound bed. A donut-shaped plastic splint was placed around the wound, with the area centered within the splint and the splinted hole represented the original wound size. An immediate-bonding adhesive was used to fix splints to the skin, followed by interrupted sutures to stabilize its position. Animals were housed individually. Digital photographs of wounds were taken at days 0, 3, 7 and 12 after cell injections and the percentage of wound closure was calculated [40] as: Wound closure (%)  =  (Areas of original would – Area of actual would)/Area of original would) ×100.

Time to wound closure was defined as the time at which the wound bed was completely re-epithelialized and filled with new tissue. Mice were sacrificed at day 12, and skin samples (including the wound and 4 mm of the surrounding skin) were harvested and placed on a tissue culture dish with the dermis side down, and photographed immediately.

#### Conditioned media (CM)

CM were obtained from confluent hWMSCs or MSC-End30d cultured for 48 h in M199 medium with 10% FBS. Cells and debris were removed by centrifugation (5 min, 500 g) and CM was stored at −80°C until use.

#### Histologic Examination

Tissue specimens were fixed (3% paraformaldehyde for 2 h) and embedded in paraffin. Six-micron-thick sections were stained with hematoxylin and eosin for light microscopy. Histological scoring was performed in a blinded fashion. The criteria used for histological scores of wound healing are summarized in Table 2, as described previously [39]. In brief, each slide was given a histological score ranging from 1 to 10 according to the following parameters: re-epithelialization and regeneration, dermal cellularity, granulation tissue formation, and angiogenesis. Capillary density was assessed morphometrically by examining three fields per section of the wound between the edges in six successive sections after measuring by Image Pro plus software (Ipwin32, American, USA) [34], [39].

**Table 2:** Criteria for histological scores.

Score	Epidermal and dermal regeneration	Cell infiltration	Granulation tissue	Angiogenesis
1–3	Minimal to moderate re-epithelialization with or without minimal developing glandular structure formation in the wound	Wound covered with thin to moderate cell layer	Granulation around wound edges only	Capillary density <400/mm2
4–7	Complete re-epithelialization with minimal developing glandular structure formation in the wound	Wound covered with thick cell layer	Granulation around wound edge and in 30%–50% of wound bed	Capillary density 400–600/mm2
8–10	Complete re-epithelialization with considerable developing glandular structure formation in the wound	Wound covered with very thick and densely populated cell layer	Thick granulation around wound edge and in >50% of wound bed	Capillary density >600/mm2

#### Microscopic analysis and immunohistochemistry

Paraffin-embedded tissue sections were cut into 4-mm slices to use for vessel identification as previously described [38], [39]. This approach, allowed us to measure the perimeter of every vessel and its corresponding total area of the reference field. These were expressed as mean vessel perimeter, in micrometers, divided by the respective total area. The blood vessels area in the pictures was analyzed using ImageJ software (National Institutes of Health, Bethesda, MD).

#### Presence of hWMSCs into the healing area

hWMSCs were identified by human mitochondrial immunohistochemistry using same approach as described above. Sections were reacted with anti-human mitochondria (1∶1000, Abcam), and visualized with a peroxidase-conjugated secondary antibody (1∶100, Sigma Aldrich). Five fields belong to 5 tissue sections per animal were randomly selected. Positive cells in each field was evaluated as a dichotomic variable (i.e, present or absent). All analysis were carried out in a blinded way.

### Statistical analysis

Values were expressed as mean ± standard deviation where *n* indicates number of different cell cultures (in duplicate). Statistical analysis of data was performed with a one-way ANOVA followed by a Tukey–Kramer. We used *X* ^2^ test to analyze proportions. The statistical software GraphPad Prism 5.00 (GraphPad Software Inc., California, USA) and standard statistical software (SPSS 10.0) were used for data analysis. Differences were considered significant when the P-value was 0.05 or less.

## Results

### Characterization of mesenchymal stem cells isolated from Wharton's jelly

**Figure 1:**
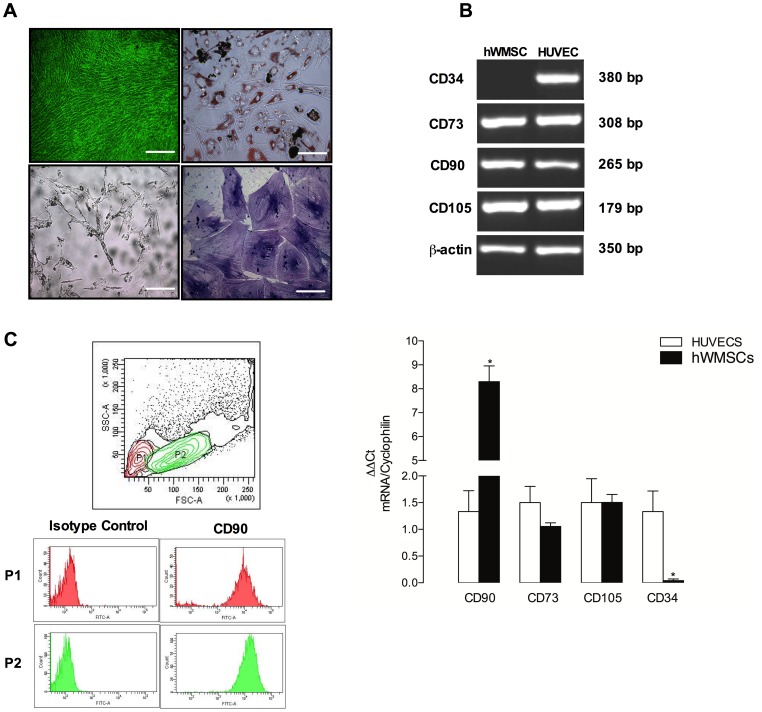
Phenotypic characterization of MSCs isolated from Wharton's jelly (hWMSC). A) hWMSCs were cultured with basal medium (upper left panel), adipogenic (upper right panel), osteogenic (lower left panel) or chondrogenic (lower right panel) medium for 10 days. Cells were stained for lipid droplets (Oil Red O staining), calcium deposits (Von Kossa staining) or sulfated proteoglycans (toluidine blue staining), respectively. Magnification 20x. B) Mesenchymal gene expression (CD91, CD73, CD105, CD34) was assessed by RT-PCR in hWMSCs cultured in non-differentiated medium. Human umbilical vein endothelial cells (HUVEC) were used as control. Lower panel: qPCR shows RNA levels for Mesenchymal gene expression in hWMSC versus the corresponding controls. C) Surface CD90 expression was evaluated on hWMSCs by flow cytometry. Data are representative of 5 different isolations of MSC from Wharton's jelly.

Isolated cells showed a spindle-shaped fibroblast-like morphology (Figure 1A). After 15 days in culture in the presence of differentiating medium, hWMSCs exhibited positive staining for osteoblasts, adipocytes and chondrocytes markers (Figure 1A).

hWMSCs cultured in non-differentiating media expressed the mesenchymal markers CD90, CD73 and CD105, as assessed by RT-PCR, but were negative for the hematopoietic marker CD34 (Figure 1B). On the contrary, CD34 mRNA level was significantly higher (7 fold) in human umbilical vein endothelial cells (HUVEC), as compared to hWMSCs (Figure 1B).

Flow cytometry analysis showed two subpopulations of hWMSC (i.e, P1, P2), which differ mainly in size. Moreover, both subpopulation were positive for CD90 (96.0% for P1 and 98.5% for P2) (Figure 1C). In addition, using flow cytometry, we also observed that hWMSCs were positive for vimentin (95%), but showed a low expression of the endothelial markers CD31 and KDR (data non show).

#### Characterization of hWMSCs trans-differentiated to endothelial cells

**Figure 2:**
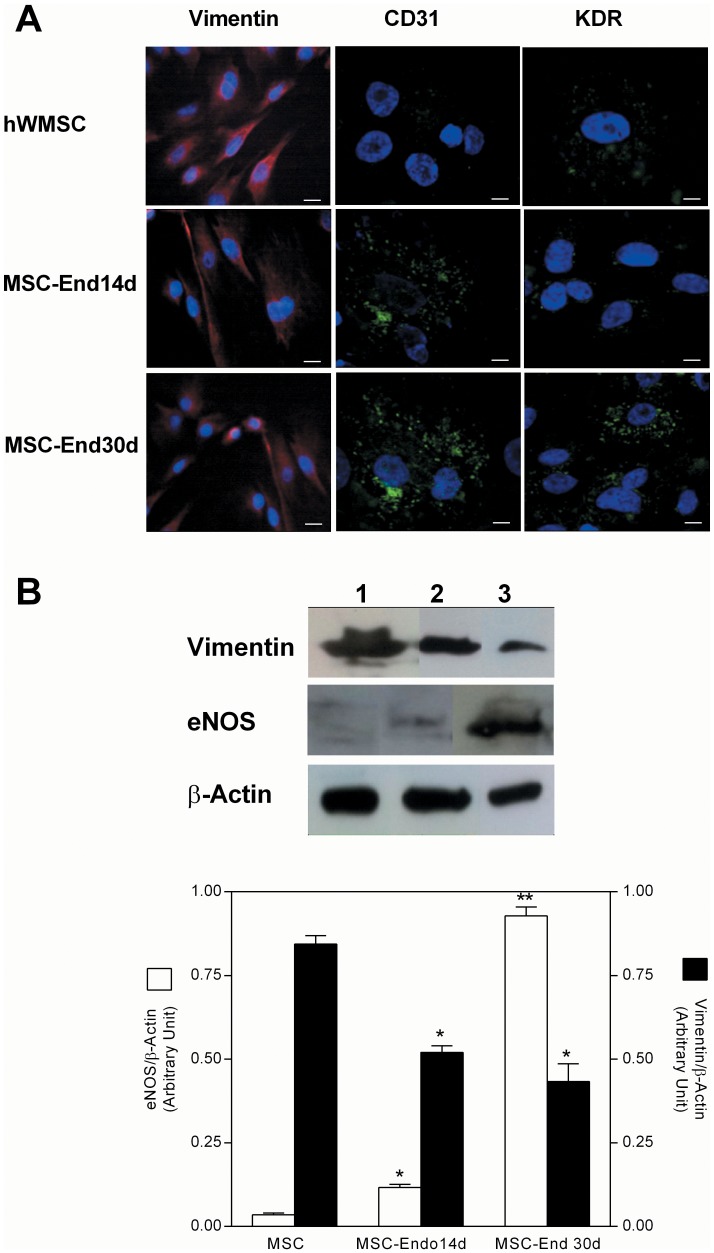
Endothelial trans-differentiation of mesenchymal cells isolated from human Wharton's jelly. A) Confocal microscopy for vimentin (mesenchymal cell marker), KDR and CD31 (endothelial markers) by immunofluorescent staining. Normal mouse IgG was used as a negative control. Nuclei were counter-stained with DAPI (blue). Magnification 40X (CD31 and KDR), 20X (vimentin). Bars 50 µm. B) Vimentin and eNOS protein abundance in hWMSCs (line 1) or hWMSC transdifferentiated into endothelial cells for 14 days (hWMSC-End14d, line 2) or 30 days (hWMSC-End30d, line 3) with endothelial-differentiating medium. Upper panel: Representative western blot for vimentin, eNOS and β-actin (internal reference). Lower panel: densitometric analysis of protein abundance, expressed as ratios for Vimentin/β-actin (**▪**) or eNOS/β-actin (**□**). Data are representative of 5 different MSC isolations from Wharton's jelly.

Using both inmmunocytochemistry (Figure 2A) and western blot analysis (Figure 2B), it was observed that after 15 days of culture without differentiating medium hWMSCs expressed high protein levels of vimentin protein, but little eNOS, CD31 and KDR levels. On the contrary, in the presence of culture medium for endothelial differentiation (see Methods), hWMSC-End14d and hWMSC-End30d showed low levels of vimentin, but high levels of eNOS, CD31 and KDR. In addition, expression of eNOS protein (an endothelial marker) was higher (4 fold) in hWMSC-End30d compared with MSC-End14d (Figure 2B).

**Figure 3:**
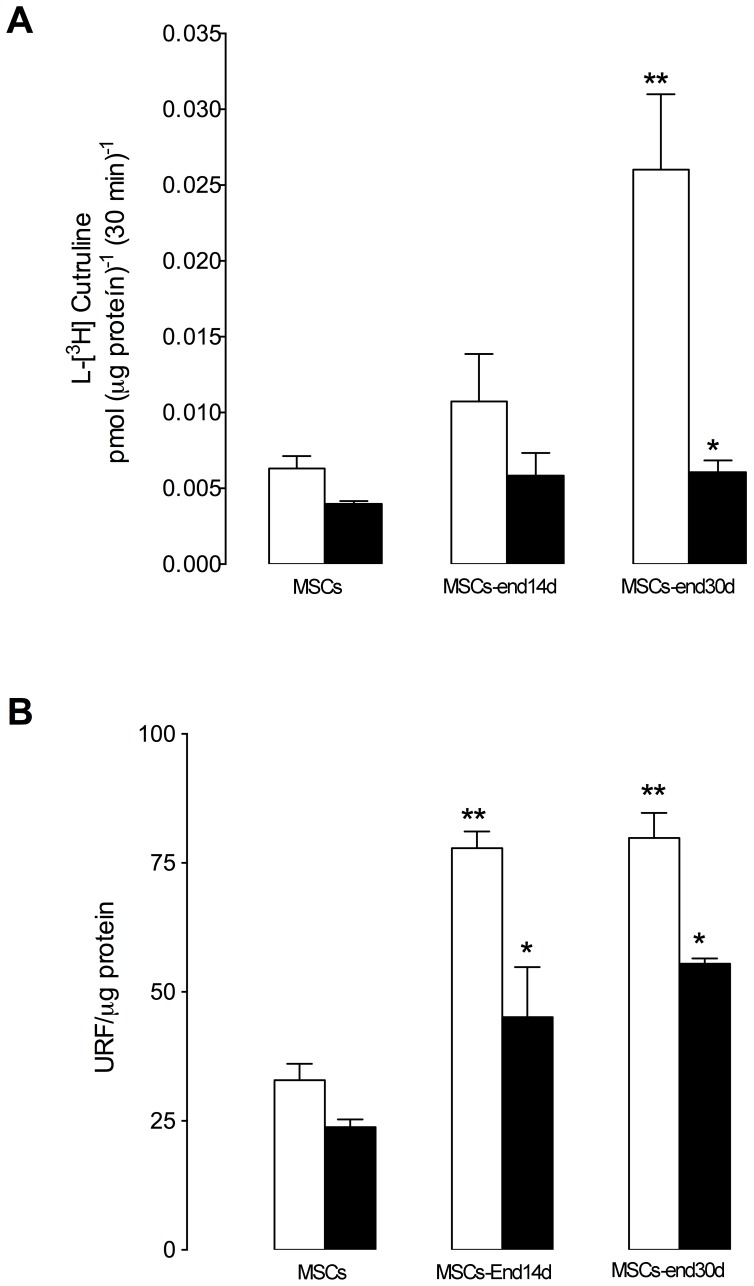
L-citrulline formation and NO bioavailability in endothelial-transdifferentiated mesenchymal cells isolated from human Wharton's jelly. NO synthesis was estimated in hWMSCs or hWMSC transdifferentiated into endothelial cells for 14 days (hWMSC-End14d) or 30 days (hWMSC-End30d). Cells were incubated in the absence (control □) or presence of L-nitroarginine methyl ester (L-NAME, 100 mM, ▪) for 30 minutes. A) L-citrulline formation assay; B), NO detection with the 4,5-diaminofluorescein diacetate probe (DAF-FM-DA, 1 mM, 30 minutes). Values are mean±S.E.M (n = 3), **P<0.01 v/s MSCs; *P<0.05 v/s without L-NAME. Data are representative of 5 different MSC isolations from Wharton's jelly.

Conversion of L-arginine to L-citrulline (i.e., eNOS activity) and intracellular NO formation were elevated (5 and 3 fold, respectively) in trans-differentiated endothelial cells, when compared with non-differentiated cells. Indeed, NOS activity was higher (2 fold) in hWMSC-End30d than hWMSC-End14d (Figure 3A); whereas NO intracellular formation reached the same level in both stages of differentiation. Both L-citrulline formation and NO formation were inhibited by L-NAME in all conditions (Figure 3).

#### Wound healing capacity of endothelial-trans differentiated hWMSCs

**Figure 4:**
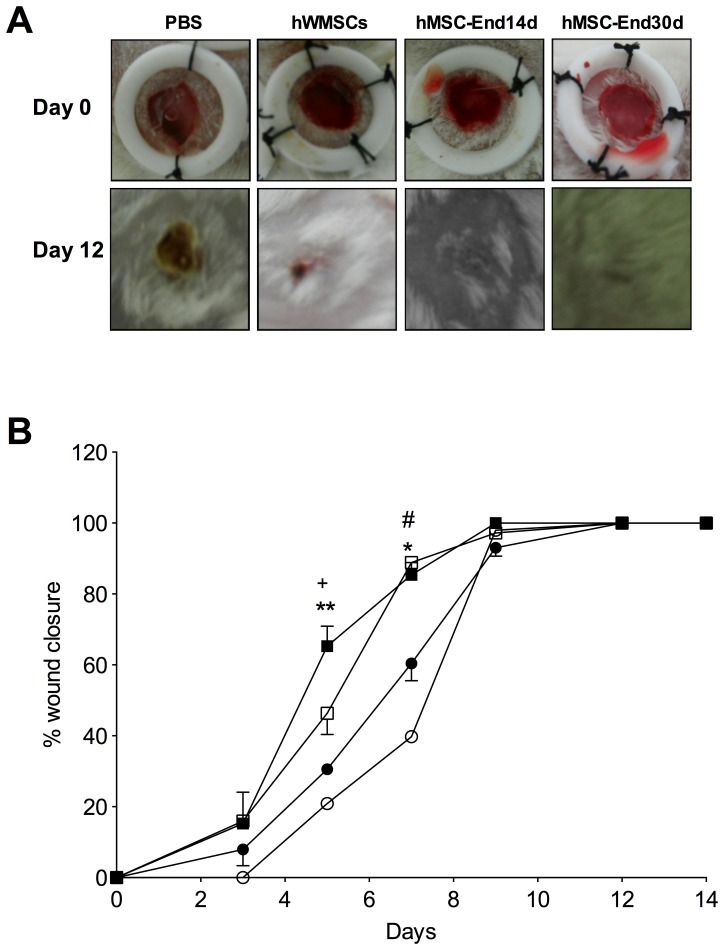
Effect of hWMSCs and endothelial-differentiated hWMSC transplantation in a wound-healing model. **A**) Representative images of wounds at day 1 (top panels) and 12 (lower panels) after injury and subcutaneous injection of hWMSCs, hWMSC trans-differentiated into endothelial cells for 14 days (hWMSC-End14d) or 30 days (hWMSC-End30d), or control (PBS). **B**) Wound healing quantified in PBS (○), hWMSC (•), hWMSC-End14d (□) or hWMSC-End30d (▪) treated mice (n = 5 independent experiments, in duplicate). Values are expressed as mean±S.E.M, ^+^P<0.05 in hWMSC-End30d v/s hWMSC, hWMSC-End14d, at the corresponding time; **P<0.03 in hWMSC-End30d v/s PBS; *P<0.001 in hWMSC-End30d v/s PBS; # P<0.01 in hWMSC-End30d v/s PBS.

From hWMSC was possible to obtain trans-differentiated endothelial cells of 14 (MSC-End14d) and 30 day (MSC-End30d). Thus, three cell populations were obtained from the same hWMSC isolation, each one was injected in two animals, for wound healing experiments (n = 5 experiments). Mice treated with hWMSCs exhibited accelerated wound closure as compared to control animals (Figure 4A). Among hWMSC, those cells that were trans differentiated toward an endothelial phenotype showed faster wound closure than non-differentiated hWMSC. Thus, at day 7, the extent of wound closure was higher in mice treated with hWMSC-End14d and hWMSC-End30d (90±3%) compared to those mice that received either hWMSCs (55±4%, p<0.05) or vehicle (39±1%, n = 5, p<0.05). Similarly, complete wound closure was observed at day 12 in the hWMSC group, but at this time wound closure remain incomplete (97±2%) in mice treated with vehicle (Figure 4B).

**Figure 5:**
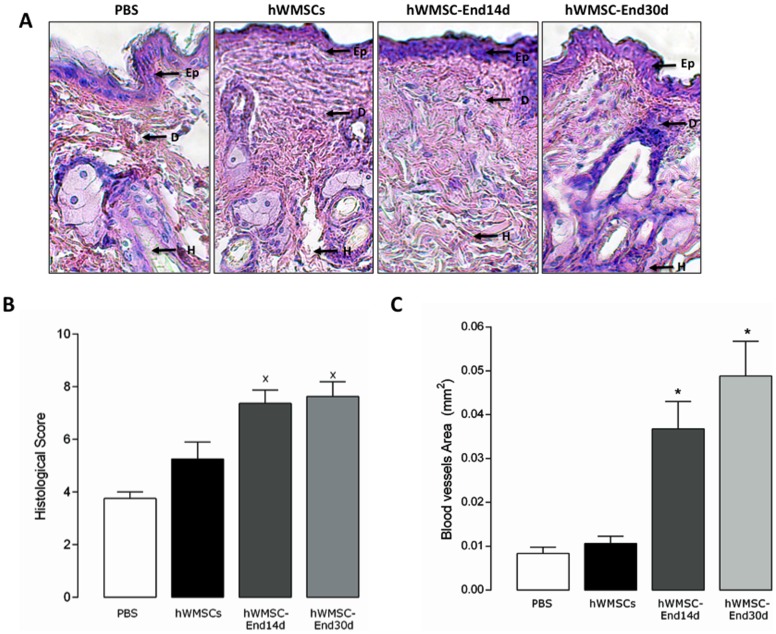
Histologic analysis of wounds in the wound-healing model. **A**) Representative photographs of wounds (hematoxilin/eosin staining) 12 days after injury and subcutaneous injection of PBS, hWMSCs, hWMSC-End14d or hWMSC-End30d. Quantification of histological images, for blood vessels area (B) and histological score (C) for each group of mice. Values are mean ± S.E.M (n = 5 independent experiments, in duplicate), *P<0.001 in hWMSC-End30d or hWMSC-End14d v/s MSC; ^+^P<0.05 in hWMSC-End30d or hWMSC-End14d v/s hWMSC. Magnification x40 (-). Ep, epidermis; D, dermis; H, hypodermis.

Histological analysis of the healed tissue at day 12 showed enhanced re-epithelialization in mice treated with hWMSC-End14d and hWMSC-End30d compared with those treated with either hWMSCs or vehicle (Figure 5A). Indeed, in animals treated with hWMSCs, hWMSC-End14d or hWMSC-End30d, wounds exhibited augmented thickness of the epidermis, newly formed granular, tissue, and a more organized extracellular matrix (Figure 5A), as well as increased area and number of vessels (Figure 5B), but the effect only reached statistical significance in endothelial-trans differentiated hWMSC (14d or 30d) compared with the vehicle-treated group.

Consistent with these findings, the histological score, an index of repairing capacity, was significantly higher in the groups treated with hWMSC-End14d and hWMSC-End30d compared with hWMSC or vehicle (Figure 5C). There was no significant difference among histological parameters when comparing histological scores in the hWMSC-End14d and hWMSC-End30d groups.

#### Engraftment of endothelial-trans differentiated hWMSCs into wounded skin

**Figure 6:**
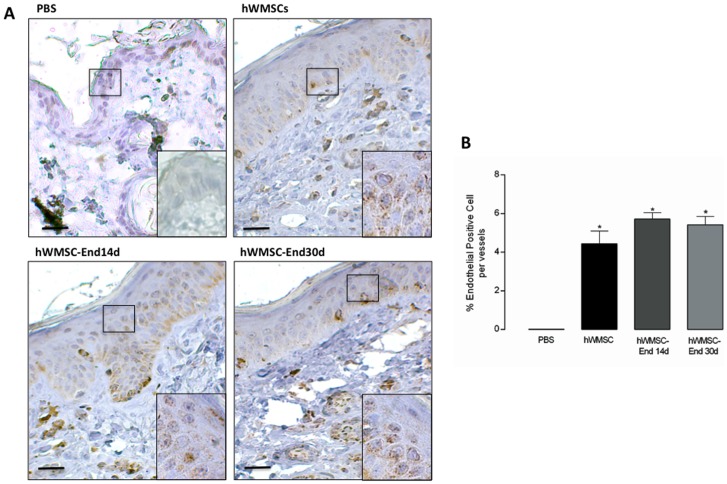
Immunohistochemical detection of human mesenchymal cells in a wound-healing model. **A.** Immunohistochemical staining of human mitochondria was performed in permeabilized tissue sections obtained after 12 days of subcutaneous injection of PBS, hWMSCs, hWMSC-End14d or hWMSC-End30d in mice. Cell nuclei were stained with hematoxyline. In B. Number of positive cells per vessel. Representative images of 5 independent experiments, in duplicate. Magnification x40 and insert 100x. Bars 50 µm.

Positive staining for human mitochondria was found in scar tissues in the three groups of animals (hWMSC, hWMSC-14d and hWMSC-30d treated mice), being the hWMSC-30d group the most evident. Positive staining was localized in epidermis, dermis, hair follicles and glandular structures in all grafted animals, in contrast, PBS-treated mice were completely negative for human mitochondrial staining (Figure 6A). The number of positive cells observed in vessels is restricted and there are no significant differences in different types of cells implanted. (Figure 6B)

#### Wound healing with conditioned medium

Conditioned medium (CM) recovered from hWMSCs or hWMSC-End30d cultures increased the formation of capillary-like structures in HUVEC cultured on Matrigel. Thus, when compared with medium derived from hWMSCs, the conditioned medium from hWMSC-End30d significantly increased length, number of branch points and relative area of capillary-like structures in HUVEC cultured on Matrigel (data not show).

**Figure 7:**
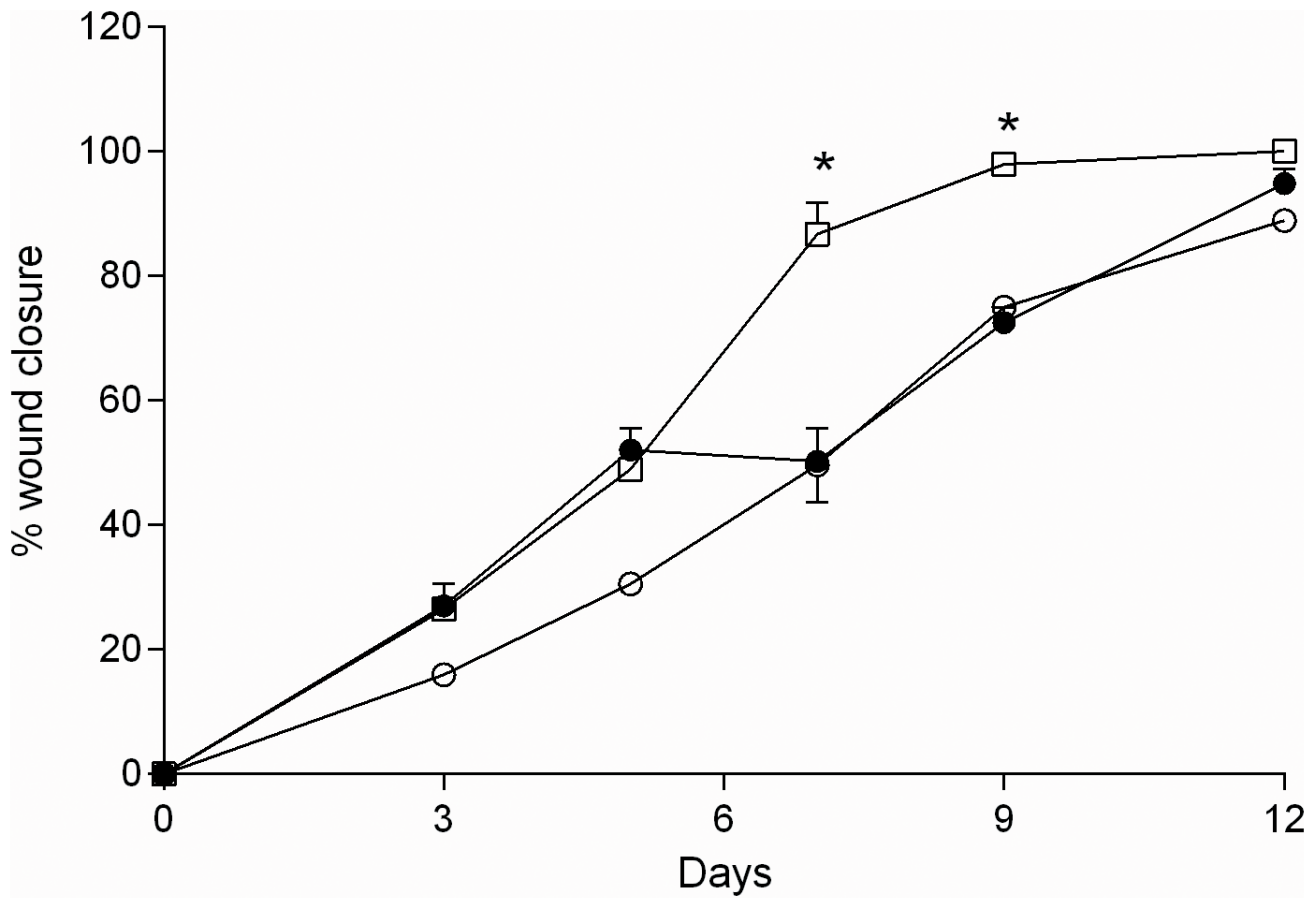
Effect of conditioned medium on wound repair. Wound closure was quantified in injured mice that received an injection of non-conditioned medium (○), conditioned medium derived from hWMSC (•) or hWMSC-End30d (□) (n = 5 experiments in duplicate). Values are expressed as mean±S.E.M, *p<0.04 vs non-conditioned and conditioned medium derived from hWMSC.

When injecting CM directly on wounds in the animal model of injury, there was a significant increase in the percentage of wound closure, from day 7 to day 9, in mice treated with hWMSC-30d medium compared with hWMSCs medium or PBS (p<0.05) (Figure 7).

## Discussion

Mesenchymal stem cells isolated from Wharton's jelly of human umbilical cords (hWMSCs) have been proposed as an alternative source of progenitor cells for use in regenerative medicine [17], [41]–[44]. On the other hand, loss of endothelial cells or endothelial dysfunction is associated to several chronic metabolic diseases. Endothelial repair using adult cells is extremely difficult, since their isolation from large vessels is challenging, and once isolated they display a low proliferation rate in culture [39]. In this regard, our results show that: 1) hWMSCs can be differentiated into adipocytes, osteocytes and chondrocytes. 2) hWMSCs express classic surface mesenchymal markers (CD90, CD73 and CD105), but not hematopoietic markers (CD34). 3) These cells can be trans-differentiated into endothelial cells (hWMSC-End), which in turn show expression of endothelial markers, such as CD31, KDR and eNOS, and importantly, they produce NO. 4) *In vivo* experiments showed that hWMSCs trans-differentiated into endothelial cells have greater regenerative capacity than non-differentiated hWMSC. 5) Conditioned medium derived from hWMSC-End, also enhanced the percentage of wound healing and vascularization in the scar, suggesting that these cells may release pro-angiogenic factors. Therefore, trans-differentiation of hWMSC into endothelial cells enhances wound healing and potentially exhibits a broad range of pre and clinical applications.

### hWMSCs characterization and endothelial differentiation

Our phenotypic characterization of hWMSCs agrees with previous literature [18]–[20], where primary cultures include at least two subpopulations of cells that differ in size: a larger (P1) and a smaller population (P2). Both cell types were positive for the mesenchymal marker CD90 (96 and 98.5%, respectively) (Figure 1C), but not for the hematopoietic marker CD34, as reported previously [43], [45], [46]. Functional studies showed that hWMSCs can be differentiated into chondrocytes, osteocytes, adipocytes, as well as they can be trans-differentiated into endothelial cells. The International Society for Cellular Therapy (ISCT) propose three criteria to define MSC include: plastic adherence in conjunction with a fibroblastoid phenotype; cell surface expression of CD105, CD73 and CD90 and lack of expression of CD45, CD34, CD14 (or CD11b), CD79α (or CD19) and human leukocyte antigen (HLA)-DR molecules; and *in vitro* differentiation capacity toward chondrocyte, adipocyte and osteocyte lineages. Our results agree with these criteria proposed by the ATCC. In addition, Lui et al., (2014) showed that the human umbilical cord Wharton's jelly is an potential alternative source of highly pure mesenchymal cells, since contrary to bone marrow or adipose tissue, the Wharton's jelly preparations contained reduced amount of stem and hemogenic cells [25].

In addition, when hWMSCs were exposed to an endothelial-differentiating medium, they showed morphological changes (polygonal-elongated morphology, enlarged nuclei and prominent nucleoli), which have been associated with hWMSCs trans-differentiation [5], [47]. Furthermore, endothelial marker expression (CD31, KDR and eNOS) increased during differentiation and was significantly higher in hWMSC-End30d than in hWMSCs or hWMSC -End14d, supporting cell differentiation towards an endothelial phenotype as demonstrated by others [31], [43], [47]. Therefore, our results further support the capacity for trans-differentiation of hWMSCs.

Alamino et al., (2010) demonstrated that specific genes related to endothelial cell phenotype and function were up-regulated in hWMSCs stimulated by endothelial differentiating medium [47]. However, despite this and the phenotypical differentiation highlighted in other studies [43], [44], [47] relevant endothelial-related functions, such as NO synthesis, had not been analyzed. Bone marrow mesenchymal stem cells can also be differentiated into endothelial-like cells [45], [46], [49], [50]. However, bone marrow harvesting is a highly invasive procedure to the donors, and proliferation efficiency, multipotent differentiation potential, and maximal lifespan of these cells decline with aging [51]. This is one of the main reasons for seeking other sources of stem cells. In this context, it has been shown that mesenchymal stem cells derived from human placenta were able to differentiate into endothelial-like cells [52], whereas differentiation capacity toward osteocytes, adipopocytes or chondrocytes was not tested. This last limitation was overcome in our study, showing that hWMSCs can be differentiated in osteocytes, adipopocytes, chondrocytes and even endothelial cells.

Mature endothelial cells are characterized by the synthesis and release of vasoactive substances, such as NO. We find that eNOS protein expression was significantly increased only in hWMSC-End30d, and effect associated with high L-arginine conversion into L-citrulline (i.e., an index of NOS activity) and intracellular formation of NO, confirming that hWMSC-End30d displays an endothelial phenotype. In this regard, the effect of NO on tissue repair is mediated by at least two of the three isoforms described, inducible nitric oxide synthase (iNOS) and eNOS, since mice deficient to those enzymes exhibited an impaired healing processes [43], [47]. Moreover, eNOS gene-transduction in mice restored the attenuated NOS production from the injured endothelium, resulting in vasodilation and regeneration [48]–[53]. The underlying mechanisms triggered by NO during tissue repair remain largely unknown. However, it has been suggested that NO regulates the expression of an increasing number of genes, including VEGF, which in turn plays a crucial role in tissue repair processes [7], [21]. In agreement with this last idea, our results suggest that hWMSCs, and particularly hWMSCs-End30d release a proangiogenenic factor(s), since the use of conditioned medium from those cells, also accelerated tissue repair compared to animals treated with vehicle or non-conditioned medium. However, to determine the precise mechanism involved, identification of these factors is required, as well as characterization of morphological and/or histochemical changes in the repaired tissue.

### Wound healing with endothelial trans-differentiated hWMSCs

Mesenchymal stem cells accelerate wound healing in animal models [35], [47]–[49]. Our results agree with these evidences, and indicate that implantation of hWMSCs differentiated into endothelial cells and cultured during 14 or 30 days showed faster wound healing than hWMSCs. The mechanisms are unclear; however, as described above we found some differences in the capacity for NO synthesis, which may impact the healing capacity of these cells. In addition, the capacity and functionality of differentiated mesenchymal stem cell might differs *in vivo* depending on the source from where they were isolated [27]. For instance, hWMSC-End, or mesenchymal stem cells isolated from amnion [38] but not bone marrow-derived cells [39] improve wound repair. More studies are required in order to confirm higher capacity of feto-placental derived mesenchymal cells compared those isolated from bone marrow or adipose tissue.

Histological analysis of scar tissue obtained from wounds showed higher capacity regeneration of hWMSC-End compared to hWMSCs. Thus, hWMSC-End treated animals showed the highest regeneration capacity, associated with better organization of epidermis and dermis compared to animals treated with hWMSC, as indicated by the high histological score observed in those animals. Interestingly, tissue recovery in the group of animals treated with hWMSC-End was associated with an elevated number of blood vessels than hWMSCs or PBS-treated animals. In this regard, neovascularization is a crucial step in the wound healing process [5], [8], [15], since this process is necessary to sustain the newly formed granulation tissue and the survival of keratinocytes.

We conduct additional experiments in order to answer whether the presence of hWMSCs or release of pro-angiogenic factors may be taken part in the tissue recovery. Firstly, we demonstrated that hWMSCs are incorporated into the scar tissue (epidermis, dermis, hair follicles and glandular structures), a finding more evident when hWMSC-End30d were used. These results are consistent with previous studies where bone marrow derived mesenchymal stem cells were incorporated into hair follicles and sebaceous glands in a mouse model of injury [39], [54]. However, the number of human cells identified in the lesion area was reduced, as previously reported using mesenchymal stem cells isolated from mouse bone marrow [27]. In addition, a recent study showed a dramatic decline of engrafted bone marrow mesenchymal stem cells in the acutely infarcted myocardium after intramyocardial injection [55]. The mechanisms involved with the decline of implanted cells are not fully understood. It is likely that with progression of wound healing process, cytokines and extracellular matrix molecules favor both mesencymal stem cells survival and engraftment decrease. In this scenario, we further investigate whether hWMSCs and hWMSC-End may promote wound healing via soluble factors.

In order to test the last hypothesis, we recovered conditioned media from hWMSC or hWMSC-End30d cultures, which were injected into mice wounds. Interestingly, conditioned medium derived from hWMSC-End accelerated wound closure, in a similar pattern that observed with hWMSC itself (figures 4B and 7, respectively). These results, suggest that wound healing is mainly carried out by soluble factors (i.e. cytokines or growth factors) produced by hWMSC-End. Indeed, it is well known that hWMSCs may secrete angiopoietin-1, angiogenin, interleukin 8 (IL-8), monocyte chemotactic protein-1 (MCP-1), serpin E1 (plasminogen activator inhibitor 1), tissue inhibitor of metalloproteinase-1 (TIMP-1) and trombospondrina-1 [27]. A combination of those factors (and perhaps many others) may participate in the healing effect of conditioned medium derived from hWMSC-End [36]. In our study we also found that CM recovered from hWMSCs or hWMSC-End30d cultures increased the formation of capillary-like structures in HUVEC cultured on Matrigel, supporting that pro-angiogenic factor(s) might taken part in the promotion of wound healing. Alternatively, other group has suggested that mesenchymal stem cells may recruit mature endothelial cells and/or release pro-angiogenic molecules [34], which then may contribute to the healing process [56]. Clearly, more studies are required in this field.

In conclusion, our results represent the first evidence showing that endothelial-trans differentiated hWMSCs have the capacity to promote neovascularization and wound healing *in vivo* through the secretion of pro-angiogenic factors. However, further studies are required to identify whether NO or other secreted angiogenic factor(s) are responsible for tissue regeneration *in vivo*, and evaluate their future use in regenerative therapy.
